# Weight gain during pregnancy and its associated factors: A Path analysis

**DOI:** 10.1002/nop2.539

**Published:** 2020-06-17

**Authors:** Mahrokh Dolatian, Nasibeh Sharifi, Zohreh Mahmoodi, Azita Fathnezhad‐kazemi, Elahe Bahrami‐vazir, Tayebeh Rashidian

**Affiliations:** ^1^ Department of Midwifery and Reproductive Health School of Nursing and Midwifery Shahid Beheshti University of Medical Sciences Tehran Iran; ^2^ Department of Midwifery School of Nursing & Midwifery Ilam University of Medical Sciences Ilam Iran; ^3^ Social Determinants of Health Research Center Alborz University of Medical Sciences Karaj Iran; ^4^ Department of Midwifery Faculty of Nursing and Midwifery Islamic Azad University, Tabriz branch Tabriz Iran; ^5^ Department of Obstetrics and Gynecology Medical School Ilam University of Medical Sciences Ilam Iran

**Keywords:** midwifery, nurses, nursing, path analysis, pregnancy, weight gain, womens health

## Abstract

**Aim:**

Weight gain during pregnancy is an important indicator in the prediction of morbidity and mortality in infants and mothers. This study aimed to determine the association factors for weight gain during pregnancy.

**Design:**

A longitudinal study.

**Methods:**

A total of 734 women were selected using multistage cluster sampling. Data were collected using demographic and midwifery questionnaires, economic and social status, psychological factors, domestic violence, perceived social support and food insecurity.

**Results:**

Of participants 28.7%, 49.6% and 21.7%, respectively, received insufficient, adequate and excessive weight gain in pregnancy respectively. Among health determinants entered in the model, mother's age, prepartum body mass index and direct and indirect prenatal care, size of households, food insecurity, stress, anxiety, stress and pregnancy‐specific stress as well as violence had a positive and increasing effect on weight gain during pregnancy.

**Conclusion:**

Considering the effect of inappropriate weight gain during pregnancy on undesirable pregnancy outcomes, related factors such as nutritional status, stress and depression in prenatal care should be assessed. Healthcare providers should consult, educate pregnant women.

## 1 INTRODUCTION

Gestational weight gain and BMI of pregnant women are the important determinants of pregnancy outcomes, maternal and neonatal health (Frederick, Williams, Sales, Martin, & Killien, 2008). Accordingly, guidelines for prenatal care emphasize the importance of an adequate increase in maternal weight during gestation (Abrams, Carmichael, & Selvin, 1995; Brawarsky et al., 2005). Prenatal care providers are advised to evaluate maternal weight at each regularly scheduled prenatal visit, monitor progress towards meeting weight gain goals (Abrams et al., 1995; Garmendia, Mondschein, Matus, Murrugarra, & Uauy, 2017). Based on BMI, the recommended weight gains during pregnancy for lean women with a BMI of less than 18.5 is 13–18 kg, for those with a BMI of 18.5–24.9 is 11 to 16 kg. Moreover, the suggested weight gains for overweight women with BMI of 25–29.9 is considered to be 7 to 11 kg while weight gain of 5 to 9 kg is recommended for obese women with a BMI over 30 (Cunningham, Leveno, Bloom, Spong, & Dashe, 2014). Gestational weight gains above and below recommendations have been shown to be associated with prenatal adverse outcomes(Brawarsky et al., 2005), So that excessive weight gain during pregnancy is associated with poor neonatal outcomes like early asphyxia, birth injury and hypoglycaemia (Asvanarunat, 2014; Catalano & Shankar, 2017; Reynolds et al., 2013), large‐for‐gestational‐age infants, while inadequate weight gain is a major risk factor for adverse foetal/neonatal outcomes such as intrauterine growth restriction, preterm birth and low birth weight (Brawarsky et al., 2005; Rooney & Schauberger, 2002). Previous studies have identified various biological and non‐biological factors and modifiable behaviours as risk factors for excessive and inadequate gain (Brawarsky et al., 2005; Olson & Strawderman, 2003). Today, health has a broader perspective and social determinants of health(SDH) are considered as the most complex and controversial issues in the field of health policy (Marmot, Allen, Bell, Bloomer, & Goldblatt, 2012). Determinants or factors affecting health include the following: (a) Social, economic and political factors, culture and social system performance, (b) Structural factors, such as education, income, gender, ethnicity and employment status, which lead to social and economic inequalities, (c) Intermediate or intermediary factors (psychosocial factors: social support, self‐esteem, couples' relationship; Behavioural Factors: Unhealthy Behaviours (Violence and Addiction) and Health Systems (Sharifi, Dolatian, Kazemi Fath Nezhad, Pakzad, & Yadegari, 2018).The structural determining factors of inequalities at the level of health and psychosocial conditions are two important elements affecting health. Economic and social factors have an influence on the psychosocial, behavioural and biological factors and ultimately affect the quality of health status (Marmot et al., 2012). With a more comprehensive look at health determinants, it turns out that the problem of weight gain during pregnancy depends on several factors (Garza, 2006). Also, changes in lifestyle, nutritional behaviours and reduced physical activity have increased the prevalence of obesity (Chu, Kim, & Bish, 2009; Fathnezhad‐Kazemi & Hajian, 2019). In addition, according to the study of Davis and et al, chronic stress leads to change biological behaviours, which is followed by an imbalance diet, excessive weight gain during pregnancy and postpartum obesity(Davis, Stange, & Horwitz, 2012). Also, the results of other studies suggest that psychological factors such as stress, depression, anxiety, a history of previous psychological problems and social support are associated with being overweight during pregnancy(Mehta, Siega‐Riz, & Herring, 2011; de Rooij, Schene, Phillips, & Roseboom, 2010) and several studies have confirmed a link between household food insecurity and overweight, especially in women(Holben & Pheley, 2006; Martin & Ferris, 2007). Understanding the relative contribution of each group of factors to the risk of excessive and inadequate gestational weight gain is important in identifying at‐risk pregnant women and designing effective interventions. Considering the importance of weight gain during pregnancy, the unknown role of its associated factors, because of the lack adequate information in Ilam province, the present study was conducted to determine the gestational weight gain status and association factors with considering Social Determinants of Health, so the key factors were identified based on reviewing literature and the conceptual framework of the WHO Commission on Social Determinants of Health (SDH), study's model was designed to investigate the factors affecting weight gain during pregnancy.

## 2 MATERIALS AND METHODS

### 2.1 Study design and sampling criteria

This longitudinal study was conducted with 734 women in Ilam province On August 2016 ‐ June 2017. The inclusion criteria were, Iranian nationality, ability to read and write, singleton pregnancy, gestational age of 24–28 weeks, absence of medical problems and satisfaction for participation in the study. Individuals were excluded from the research in case of lack of collaboration to participate in the study or completion of research questionnaires. And because this second trimester is safer than other trimesters and all pregnant women go to health centres at this time to get care and deliver test results, this time was chosen to complete the questionnaires. It should be noted all health records (such as test results, weight gain at any time of pregnancy,) were recorded from the beginning until the end of pregnancy.

### 2.2 Sampling and sample size

Sampling was performed using cluster random sampling. Ilam province in southwest of Iran has 10 cities. All cities in Ilam province were divided in five geographic regions (Central, North, South, East and West) which each zone was considered as a cluster that consisted of 3 or 4 health centres. We selected one urban health per cluster by using Randomizer software. Then, family health records were reviewed, and pregnant women were identified who had 24–28 gestational weeks and were selected randomly by using Randomizer software. Initially, they were called and invited to participate in the study. In case of not participating in the first study session, or not met other inclusion criteria, other eligible participants were requested to join the study according to the list to complete the final sample size. After explaining the research aims and confidentiality of information, informed consent was obtained from the samples and the questionnaires were completed by the participants. Participants completed the questionnaires in a meeting room at the health centre for 30 min. Due to a large number of questionnaires, completing of them lasted from 20 to 35 min.

### 2.3 Measurement tools

Data were collected by questionnaires of demographic and midwifery profiles, structural health determinants (socioeconomic status), questionnaires to measure intermediate determinants of health including food insecurity, social support, stress, anxiety and depression, pregnancy‐specific stress and domestic violence. In the present study, the Socio‐demographic and obstetrics characteristics questionnaire was designed by the researcher. Validity and reliability of other questionnaire have been investigated in Iran and are available in Persian.

### 2.4 Socio‐Demographic and obstetrics questionnaire

The questionnaire was designed by the researcher and included questions about the age of the pregnant woman, the age of the spouse, the ethnicity, the gestational age, the number of pregnancies, the interval between pregnancies, the status of incidence of chronic diseases (diabetes, hypertension), consumption of supplements and so on. The section of economic and social status consists of the level of education of the pregnant mother, husband's education, woman's occupation, husband's job, number of people living at home, monthly household income, etc., the method of face and content validity was used to determine the validity of the questionnaire.

### 2.5 DASS‐21 standard questionnaire of stress, anxiety and depression

This questionnaire was designed by Lovibond in 1995 to measure each of the symptoms of stress, anxiety and depression with 7 questions in each area and Likert scale. The lowest and highest scores for each question are zero and 3, respectively. It has been used in various researches both inside and outside the country, and its validity and reliability have been confirmed and the reliability of questionnaire in the domain of depression 0.95, in the anxiety domain 0.91 and in the stress domain 0.93, with overall score of 0.97 (Sahebi, Asghari, & Salari, 2005).

### 2.6 Pregnancy‐specific stress questionnaire

The questionnaire consists of 25 phrases in 6 subsets, including maternal, infant, childbirth and motherhood experience, child and maternal, personal‐family and personal‐occupational interests. The questionnaire has been designed based on Likert scale of 5 options with a minimum and maximum scores of 0 and of 100, respectively. The score of the questionnaire indicates the level of concern of pregnant women and its worrying factors. Validity and reliability in Iran have been confirmed by Navidpour et al.(Navidpour, Dolatian, Yaghmaei, Majd, & Hashemi, 2015).

### 2.7 Domestic violence questionnaire

The World Health Organization (WHO) has designed a questionnaire to measure the violence of a spouse in pregnancy and the rate of physical, psychological or sexual violence is calculated based on the Likert scale of 5 states. The questionnaire assesses physical, sexual and emotional violence with 10, 5 and 11 questions, respectively. Individuals who have at least one positive answer to any of the questionnaire questions are considered to be violent. The Cronbach's alpha coefficient of the questionnaire in Iran has been calculated to be 0.92, 0.89, 0.88 for the three questionnaire areas of the physical, psychological and sexual violence (Hajian, Vakilian, Najm‐abadi, Hajian, & Jalalian, 2014).

### 2.8 Multidimensional perceived social support scale (MSPSS)

MSPSS was prepared by Zimatt et al. in 1998 to assess perceived social support from three sources of family, friends and important individuals in life, with a minimum score of 12 and a maximum score of 84.

Scores between 12 and 48 indicated low social support while scores of 49–68 represented the average social support level and scores of 69–84 demonstrated high social support levels; Validity in Iran was determined by content analysis, and reliability was calculated with Cronbach's alpha coefficient between 0.86 and 0.9 for subscales and 0.86 for the total instrument (Bagherian‐Sararoudi, Hajian, Ehsan, Sarafraz, & Zimet, 2013).

### 2.9 Household food insecurity access scale (HFIAS)

HFIAS consists of 9 questions and 4 options in the frequency of occurrence (most often, sometimes, rarely and never) which provided information on food insecurity in terms of access to food at the household level. The lowest score for each question was zero while the highest score for each one was 3. The validity of this questionnaire was verified by Mohammadi et al. using face, content and structure validity. Cronbach's alpha of 0.86 in the questionnaire shows its high internal stability (Mohammadi et al., 2012).

Reliability of the questionnaire was determined by using test–retest method after conducting a pilot study on 30 pregnant women. Internal consistency (Cronbach's alpha coefficient) was determined for DASS‐21 standard questionnaire, it was 0.89, 0.92 and 0.88 for domain of depression, anxiety and stress respectively and Cronbach's alpha coefficient was 0.88, 0.86, 0.88, 0.89 for pregnancy‐specific stress questionnaire, domestic violence questionnaire, multidimensional perceived social support scale and household food insecurity access scale, respectively.

**Figure 1:**
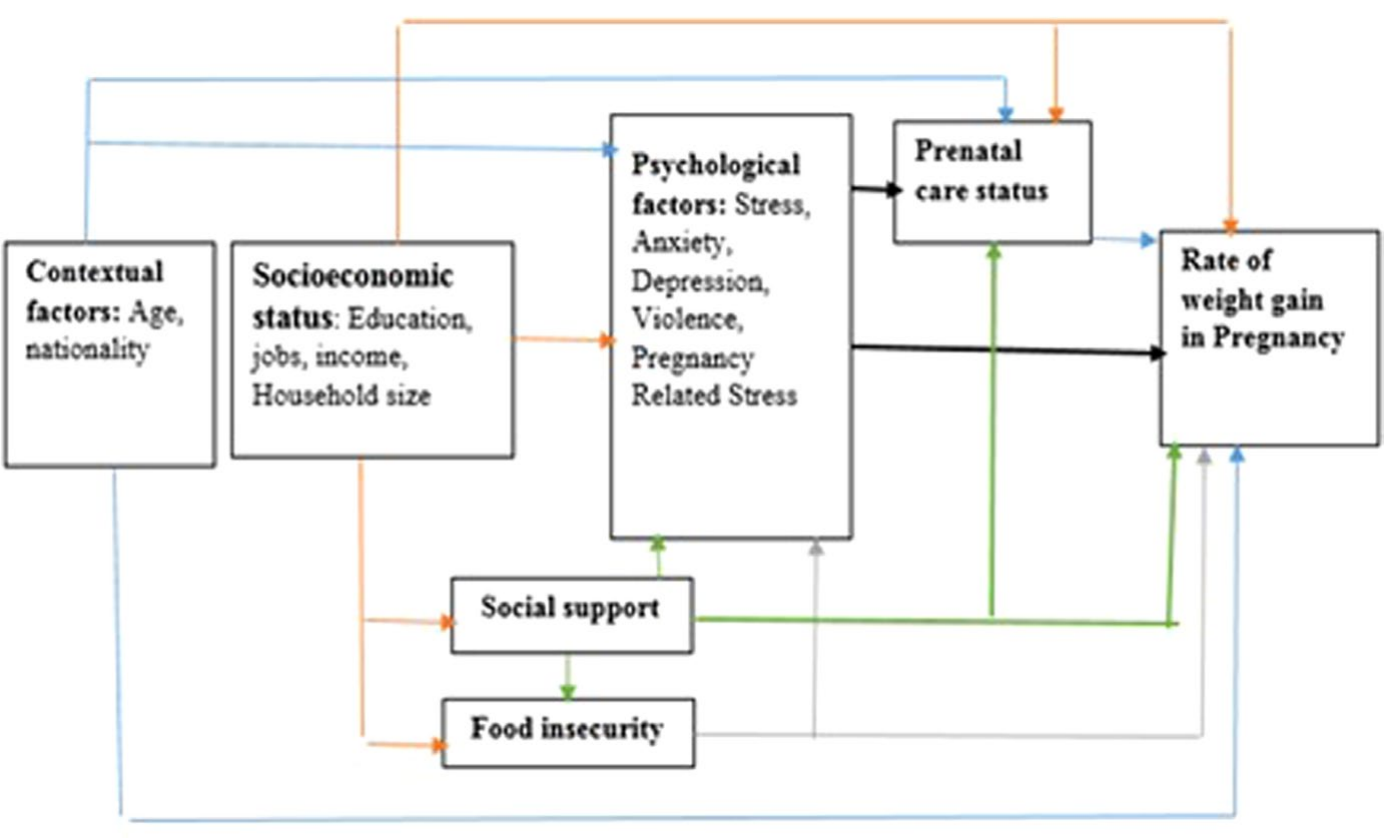
Conceptual framework. Factors associated with weight gain during pregnancy

In the current study, a conceptual model was designed to determine associated factors with weight gain during pregnancy; factors such as contextual factors, socioeconomic status, food insecurity, perceived social support, domestic Violence, stress and anxiety and depression and stressful life events, pregnancy care (Figure 1). Path analysis method was used to examine model good fit and also percentage coverage of variance.

### 2.10 Statistical analysis

After collecting data, SPSS19 software (IBM© SPSS© Statistics version 19 IBM© Corp.) and Lisrel 8.8 were used to analyse the data. Descriptive statistics were used to determine the frequency, percentage, mean and standard deviation. Chi‐square and Fisher's analytical statistics were used to assess the relationship between determinants and weight gain. Fisher statistical test was used if an expected count in the table was less than 5. Also, the path analysis was used to determine the most important variable affecting weight gain and to specify the direct and indirect effects of variables.

## 3 RESULTS

The mean age of the samples was 28.7 (*SD* 4.4) years, and the mean age of the spouses was 33.4 (*SD* 5.5) years. All samples were Iranian, 81.6% of the participants had Kurdish ethnicities. 46.3% of the participants had their first pregnancy, and 86.6% of them had wanted pregnancy and 13.4% of pregnancies were unwanted or unplanned.

About half of the samples (50.7%) and their spouses (48.2%) had university education. Most women were housewives (86.2%), and more than half of their spouses (64%) were self‐employed. In addition, the number of employees in each family was one individual in most of the families (86. 8%). The income of half of households (57.4%) was 10–20 million Rials (Table 1).

**Table 1:** The demographic and socio‐economic characteristics of the participants

Variable	Mean (*SD*)
Women's age	28.73 ± 4.41
Husband's age	33.41 ± 5.47
Variable	Number (Percent)
Type of pregnancy	Wanted pregnancy	636 (86.6)
	Unwanted pregnancy	98 (13.4)
Number of pregnancy	One	340 (46.3)
	Two	238 (32.4)
	Three and more	156 (21.3)
Ethnicity	Kurdish	599 (81.6)
	Lor	77 (10.5)
	Lak	39 (5.3)
	Others	19 (2.6)
Women's education	Elementary	29 (4)
	Secondary	49 (6.7)
	High school	284 (38.7)
	University	372 (50.7)
Husband's education	Elementary	22 (3)
	Secondary	51 (6.9)
	High school	307 (41.8)
	University	354 (48.2)
Women's jobs	Housewives	633 (86.2)
	Employees	82 (11.2)
	self‐employed	19 (2.6)
Husband's jobs	Unemployed	12 (1.6)
	Employees	470 (64)
	self‐employed	252 (34.3)
Household income	Less than 10 million Rials	125 (17)
	10–20 million Rials	421 (57.4)
	More than 20 million Rials	88 (25.6)
Number of family members	1–3	566 (77.2)
	4 and above	168 (21.9)

In the present study, 2.8% of the samples were lean, about half of women (57.7%) had normal BMI and about one third of women (39.6%) had pre‐pregnancy overweight. The mean weight gain in the study was 11.27 ± 3.23. Approximately one third of women (28.7%) had insufficient weight gain, half of women (49.6%) had enough weight gain and 21.7% had excess weight gain. Due to investigation of the relationship between structural determinants and weight gain, there was no significant difference among women's education, their husbands’ education, women's occupation, husband's job, household income, average household cost and household size in three groups (*p* > .05) (Table 2). In examining the relationship between intermediate determinants and weight gain in pregnancy, it was observed that there was a significant difference among food insecurity, social support and prenatal care in three groups of insufficient weight gain, sufficient weight gain and excess weight gain (*p* < .05).

**Table 2:** Relationship between pregnant women's socioeconomic status and weight gain during pregnancy

Variable	Low rate of weight gain Number (%)	Normal weight gain Number (%)	Excessive weight gain Number (%)	*p*‐value
Individual education				
Secondary school and lower	27 (36.5)	34 (45.9)	13 (17.6)	.182
High school education	76 (28.0)	126 (46.5)	69 (25.5)	
University	99 (27.5)	190 (52.8)	71 (19.7)	
Husband's education				
Secondary school and lower	23 (33.3)	31 (44.9)	15 (21.7)	.583
High school education	88 (30.2)	146 (50.2)	57 (19.6)	
Uniiversity	91 (26.4)	173 (50.1)	81 (23.5)	
Individual's occupation				
Housewife	179 (29.4)	294 (48.4)	135 (22.2)	.389
Employee	20 (25.6)	45 (57.7)	13 (26.7)	
Self‐employed	3 (15.8)	11 (57.9)	5 (26.3)	
Husband's occupation				
Unemployed	4 (33.3)	8 (66.7)	0 (0.0)	.151
Self‐employed	138 (30.9)	213 (47.7)	96 (21.5)	
Employee	60 (24.4)	129 (52.4)	57 (23.2)	
Household income				
Less than 10 million Rials	38 (31.9)	57 (47.9)	24 (20.2)	.082
10–20 million Rials	126 (31.3)	196 (48.6)	81 (20.1)	
Above 20 million Rials	38 (20.8)	97 (53.0)	48 (26.2)	
Average household cost				
Less than 10 million Rials	101 (29.0)	174 (50.0)	73 (21.0)	574
10–20 million Rials	96 (28.7)	167 (49.9)	72 (21.5)	
Above 20 million Rials	5 (22.7)	9 (40.9)	8 (36.4)	
Household size				
1–3 people	159 (29.3)	274 (50.5)	110 (20.3)	.233
4 people and more	43 (26.5)	76 (46.9)	43 (26.5)	

People with food insecurity had too much weight gain in pregnancy. Sufficient and high levels of overweight in pregnancy were also found in pregnant women with high social support. Also, there was no significant difference in the general violence among three groups of people with insufficient weight gain, sufficient weight gain and overweight (*p* = .99) (Table 3).

**Table 3:** Relationship between intermediate health determinants in pregnant women and weight gain in pregnancy

Intermediate determinants	Low rate of weight gain Number (percent)	normal weight gain Number (percent)	excessive weight gain Number (percent)	*p*‐value
Food security				
Has it	114 (24.9)	243 (53.1)	101 (22.1)	.008
Does not have	88 (35.6)	107 (43.3)	52 (21.1)	
Social support				
Low	29 (26.9)	59 (54.6)	20 (18.5)	.007
Average	108 (30.3)	154 (43.3)	94 (26.4)	
High	65 (27.0)	137 (56.8)	39 (16.2)	
Stress symptoms				
Does not have	150 (28.1)	268 (50.3)	115 (21.6)	.822
Has it	52 (30.2)	82 (47.7)	38 (22.1)	
Anxiety symptoms				
Does not have	169 (28.3)	298 (49.8)	131 (21.9)	.854
Has it	33 (30.8)	52 (48.6)	22 (20.6)	
Depression symptoms				
Does not have	170 (28.2)	305 (50.7)	127 (21.1)	.406
Has it	32 (31.1)	45 (43.7)	26 (25.2)	
Pregnancy‐specific tension and worry				
Does not have	148 (28.4)	270 (51.7)	104 (19.9)	.093
Has it	54 (29.5)	80 (43.7)	49 (26.8)	
Domestic violence				
Does not have	101 (26.2)	190 (49.4)	94 (24.4)	.099
Has it	101 (31.6)	160 (50.0)	59 (18.4)	
Unwanted pregnancy				
No	183 (29.8)	302 (49.2)	129 (21.0)	.178
Yes	19 (20.9)	48 (52.7)	24 (26.4)	
Pregnancy care				
Adequate	111 (21.5)	270 (52.3)	135 (26.2)	<.001
Inadequate	91 (48.1)	80 (42.3)	18 (9.5)	

**Figure 2:**
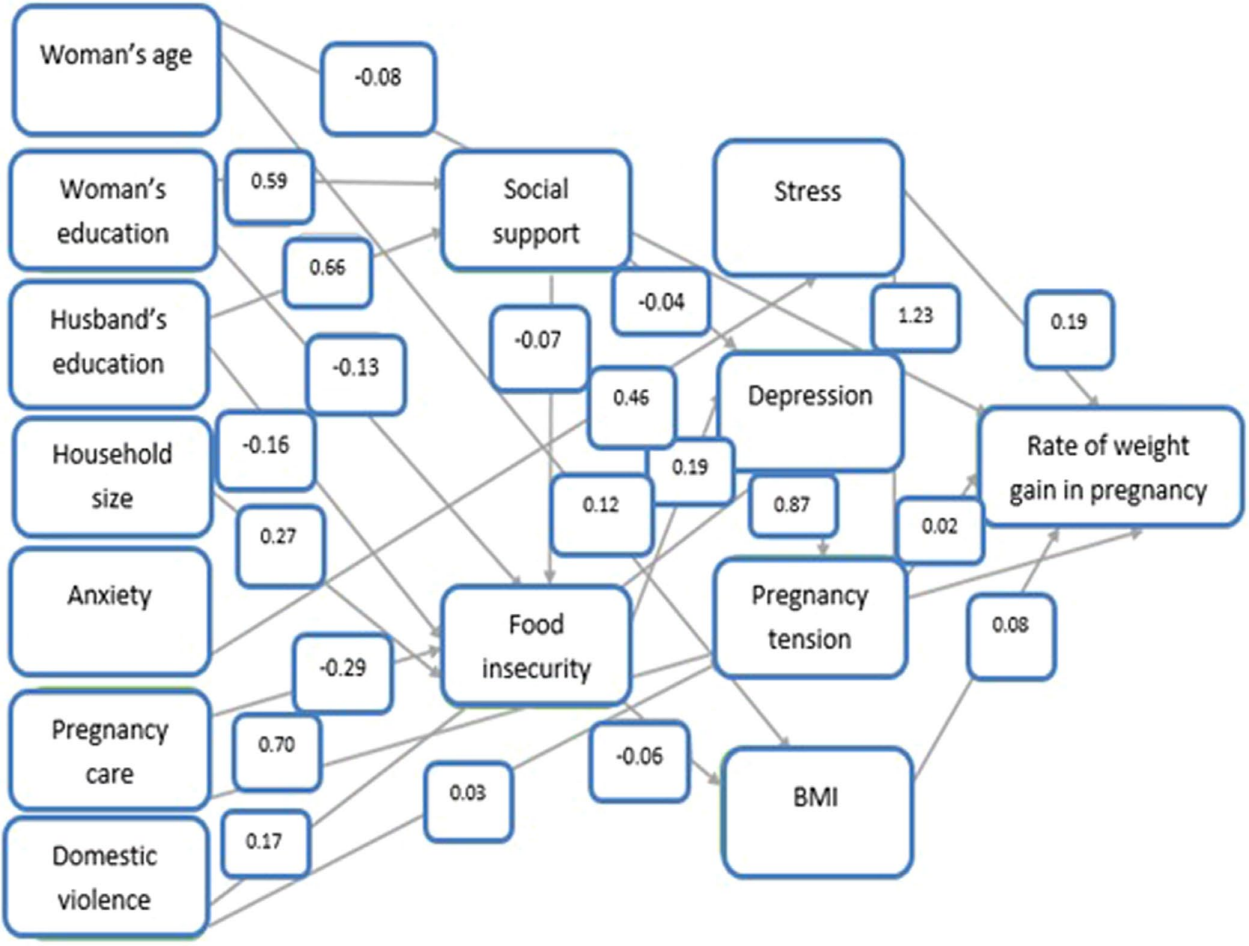
Estimated (standardized) coefficients related to the relationship between structural and intermediate factors of social determinants of health affecting on weight gain during pregnancy

Among the structural determinants, variables of mother's age, mother's education, spouse's education and household size had an impact on weight gain in pregnancy. Among these determinants, only the mother's age from direct and indirect paths had an effect on weight gain. From the direct path, mothers with older ages had reduced weight gain in pregnancy (*β =* 0.08) and from indirect path, due to the impact of mother's BMI (*β =* 0.12), BMI had a negative effect on the rate of weight gain in pregnancy (*β =* −0.08). Female and her husband's education had a negative and decreasing effect on high overweight, as the rate of overweight dropped with the increase in the level of education (Figure 2 and Table 4).

**Table 4:** Direct and indirect effects of structural and intermediate determinants of health on weight gain during pregnancy

Variable	Effects		
	Direct effect	Indirect effect	Total effect
Individual's age	−0.08	−0.010	−0.90
Individual's education	‐	−0.003	−0.003
Education of spouse	‐	−0.002	−0.002
Household size	‐	0.002	0.002
Food insecurity	‐	1.087	1.087
social support	‐	−0.002	−0.002
Stress symptoms	‐	0.025	0.025
Anxiety symptoms	‐	0.011	0.011
Depression symptoms	‐	0.031	0.031
Domestic violence	‐	0.006	0.006
pregnancy‐specific stress	‐	0.020	0.020
BMI	−0.08	‐	−0.08
status of prenatal care	0.70	−0.003	0.697

Among intermediate health determinants, two variables of mother's prepartum BMI and the condition of pregnancy cares had a direct effect on weight gain. The greatest impact was observed in the amount of pregnancy care and thus, individuals with more care had more weight gain (Table 4).

In an indirect path, food insecurity variable had the greatest impact on excessive overweight and had a positive and increasing effect on weight gain (*β* = 1.087) and excessive weight gain in pregnancy was augmented with increasing food insecurity. Food insecurity from three indirect routes had an effect on symptoms of depression, stress, tension and pregnancy‐specific concerns as well as pregnancy weight gain, and BMI contributes to excessive overweight in pregnancy (Table 4). Other intermediate determinant variables, such as symptoms of stress, anxiety, depression, pregnancy‐specific stress and violence, had an indirect effect on weight gain in pregnancy. Anxiety had a positive impact on weight gain (*β* = 0.011) due to its positive effect on stress (*β* = 0.46) as well as pregnancy‐specific tension and worry (*β* = 1.23) and the impact of worry on weight gain (*β* = 0.02). Domestic violence had an impact on weight gain from indirect path. Increased violence led to increased symptoms of depression (*β* = 0.17) and symptoms of severe depression caused an increase in stress (*β* = 0.56) as well as pregnancy‐specific anxiety (*β* = 0.87) (Figure 2). Among all the structural and intermediate health determinants entered in the model, mother's age, prenatal BMI and amount of prenatal care directly affect maternal weight gain in pregnancy while of household size, food insecurity, symptoms of stress, anxiety, pregnancy‐specific anxiety and violence had a positive and increasing effect from indirect path (Table 4). Criteria used to fit the model included goodness of fit index, normalized fit index, comparative fit index and root‐mean‐square error. In this model, criteria were appropriate, and the model fit was good (Table 5).

**Table 5:** Comparison of fitness indicators of measurement models

Index	Value
Chi‐squared index	181.14
Df	36
Root‐mean‐square error (RMSEA)	0.01
Fit goodness index (GFI)	1
Normalized fit index (NFI)	1
Comparative fit index (CFI)	0.93

## 4 DISCUSSION

In the present study, 2.8% of the samples were lean, 57.7% had normal BMI and 39.6% had prepartum overweight. In the study of Chen et al., 13.2% of women had overweight, 3.6% of them were obese and 15.8% were lean before pregnancy (Chen et al., 2010). The mean weight gain in pregnancy was 11.27 ± 3.23.

28.7%, 49.6% and 21.7% of participants respectively had insufficient, sufficient and excessive overweight in pregnancy. In the study of Deputy et al, 20.9%, 32% and 47.2% of women had insufficient, sufficient and excessive overweight in pregnancy, respectively (Deputy, Sharma, Kim, & Hinkle, 2015).

The results of present study showed that structural factors which are SDH (education of an individual, person's occupation, husband's job, household income, household size) did not have a significant relationship with weight gain but it has been specified in path analysis that the mentioned determinants have an effect on intermediate determinants of health and influence the weight gain, which is consistent with the results of the study by Finney Rutten et al. (Finney Rutten, Yaroch, Colón‐Ramos, Johnson‐Askew, & Story, 2010). In the study of Davis et al., biological, metabolic, socioeconomic and psychological factors were identified as determinants of weight gain in pregnancy (Davis et al., 2012). In the present study, rate of weight gain was higher with increasing age and prepartum BMI, which was consistent with the results of studies by Mohammadi et al., (2011); Panahandeh, (2009). In a research by Wells et al., inadequate weight gain during pregnancy was associated with underweight and obesity of mother, rural housing, low level of education, smoking and excessive weight gain while excessive overweight was related to obesity of mother and 12 years of education or less (Wells, Schwalberg, Noonan, & Gabor, 2006).

The results of path analysis showed that mother's age and education, husband's education and household size among structural determinants had an impact on overweight in pregnancy. Among these determinants, only the mother's age had an impact on weight gain both from direct and indirect paths. Individual's education and her husband's education also had a negative and decreasing effect on excessive overweight. Rate of excessive weight gain in pregnancy reduced with increased levels of education. In the study by Delaram et al., no relationship was observed between occupation of mother and overweight in pregnancy but education had a significant relation with weight gain in pregnancy and those with higher education had higher average weight gain (Delaram & Akbari, 2008). Differences in the results of the studies may be due to different sampling methods, diverse questionnaires and different classification of the variables of the questionnaire; therefore, similar studies are required to compare the results.

In the current study, a positive and significant correlation was observed between education of spouse as well as the amount of prenatal care and weight gain during pregnancy while there was a negative and significant correlation between the age of the woman, household size, pregnancy‐specific stress and prepartum BMI. In addition to demographic and structural factors of social determinants of health, intermediate factors that include stress, anxiety, depression, social support, also affect the weight gain of mother during pregnancy. In the present study, food insecurity, social support and prenatal care had a significant relationship with weight gain during pregnancy.

In the model proposed by Davis et al., maternal stress determinants included genetics, health status, ethnicity and socioeconomic status. Chronic exposure to stress caused a change in the biological behaviour leading to disturbances in maintaining balance, excessive weight gain in pregnancy and postpartum obesity (Davis et al., 2012). Studies by Bovier et al. And de Rooij et al indicated that other psychological factors such as depression, anxiety, body image dissatisfaction and social support are associated with overweight in pregnancy (Bovier, Chamot, & Perneger, 2004; de Rooij et al., 2010). The results of Herring et al., Mehta et al., showed that some maternal factors affecting weight gain in pregnancy include self‐esteem, stress, anxiety, depression and history of previous psychological problems (Herring et al., 2008; Mehta et al., 2011). In a review of Hartley et al, there was a significant relationship between pregnancy weight gain and depression as well as social support but no relationship was observed between stress, anxiety, self‐confidence and excessive weight gain (Herring et al., 2008).

Also, the results of path analysis indicated that among intermediate determinants of health, the prepartum BMI of the mother and the condition of pregnancy both had a direct effect on weight gain and the greatest impact was on the amount of pregnancy care so that those with more care had more weight gain. Other determinants also influenced the weight gain from indirect path, where the food insecurity had the greatest impact on weight gain during pregnancy. The results of present study were consistent with the outcomes of the study by Laraia et al and women with food insecurity had a higher pre‐pregnancy BMI and their weight gain was higher during pregnancy (Laraia, Siega‐Riz, & Gundersen, 2010). Studies by Martin et al. and also Holben et al. confirmed the relationship between excessive overweight and household insecurity, especially in women (Holben & Pheley, 2006; Martin & Ferris, 2007) and, consuming low‐energy foods with high energy density but low value of micronutrients is more in people with food insecurity, leading to obesity and overweight (Hill et al., 2013). One of the strengths of current study is to consider several factors of social determinants of health for the purpose of the relation assessment. Also, food insecurity is one of the most important factors that little studies have been done in this regard during pregnancy so far. Therefore, the results of present study can be used as a basis for other studies.

## 5 CONCLUSION

Given the results, half of women had enough weight gain, it seems that food security, high social support and increased the number of prenatal care are associated with a normal weight gain in pregnancy. Other variables, such as symptoms of stress, anxiety, depression, pregnancy‐specific stress and violence, had an indirect effect on weight gain in pregnancy. Therefore, the need to educate mothers about weight gain in pregnancy and its effects on inappropriate outcomes of pregnancy should be considered and its related factors such as nutritional status of pregnant women, level of stress, depression and other related factors should be investigated.

### 5.1 Limitations

The limitations of the study can be attributed to a large number of questionnaires that were taking a long time to complete the questionnaires. Using some data that contained in the prenatal care file such as weight gain in each prenatal care.

## 6 ETHICAL APPROVAL AND CONSENT TO PARTICIPATE

This study was approved by the Ethics Committee of SBMU, Grant no: SBMU.REC.1394.112 as a PhD dissertation. Written and informed consent was obtained from the participants, and they were told they could withdraw at any time during the study.

## CONFLICT OF INTEREST

No conflict of interest has been declared by the authors.

## AUTHOR CONTRIBUTIONS

All the authors contributed to the conception and design of the study. N.S wrote the first draft of the paper. M.D and A. Fn‐K and T.R revised the manuscript. Z.M and E.B have analyzed the research data. All authors read and approved the final manuscript.
